# Is There a Role for Biweekly Romiplostim in the Management of Chronic Immune Thrombocytopenia (ITP)? A Report of Three Cases

**DOI:** 10.1155/2018/6037494

**Published:** 2018-10-24

**Authors:** Jasjit Kaur Rooprai, Karima Khamisa

**Affiliations:** ^1^Faculty of Medicine, University of Ottawa, Ottawa, Ontario, Canada; ^2^Division of Hematology, Department of Medicine, The Ottawa Hospital, Ottawa, Ontario, Canada

## Abstract

Romiplostim is a peptibody, which stimulates platelet production by a mechanism similar to that of endogenous thrombopoietin. It has an established indication as second-line therapy in patients with chronic immune thrombocytopenia (ITP). The agent is typically administered weekly; however, there are instances where a biweekly (i.e., alternate week) dosing may be feasible in a select group of patients. We conducted a retrospective case review to evaluate the efficacy and safety of biweekly administration of romiplostim in maintaining a platelet count of >30 × 10^9^/L in three patients with chronic ITP. Treatment was started with a weekly injection (1 *µ*g/kg) with a dose escalation to achieve a platelet count >30 × 10^9^/L. Once stable on weekly romiplostim, these patients received biweekly administration. No bleeding complications were noted during biweekly dosing for these patients. The current findings suggest that lengthening the dose interval of romiplostim is feasible in select patients with chronic ITP to maintain stable platelet counts. Additional studies are therefore warranted to further evaluate biweekly dosing for romiplostim to increase convenience and decrease costs for patients with chronic ITP.

## 1. Introduction

Chronic idiopathic immune thrombocytopenia (ITP) is an autoimmune bleeding disorder characterized by low platelet counts often below 100 × 10^9^/L for at least 12 months' duration [1]. Recent literature suggests that the pathogenesis of immune thrombocytopenia (ITP) is caused due to both autoantibody-mediated platelet destruction and suboptimal platelet production [2–6]. Most traditional ITP therapies have focused on either inducing short-term increases in platelet counts (via intravenous immunoglobulins (IVIg), steroids, and intravenous anti-D) or long-term maintenance of platelet counts using rituximab and splenectomy [4]. These treatments were effective in many patients but failed to achieve or maintain a durable response in certain patients and were associated with adverse effects [4]. In the past decade, thrombopoietin receptor agonists (TRAs) have been shown to induce increases in platelet counts in both healthy adults and patients with ITP, with an acceptable safety profile [4–7].

Romiplostim is a thrombopoiesis-stimulating protein, referred to as a peptibody, which stimulates platelet production by a mechanism similar to that of endogenous TPO [8]. Currently, both the American Society of Hematology ITP management guidelines and the International Consensus Report guidelines recommend the use of TRAs for adults with ITP that persists following splenectomy or in patients who are not candidates for splenectomy and for whom at least one other treatment has failed [9]. In addition, the 2015 assessment report released by the European Medicines Agency (EMA) on romiplostim concluded that TRAs can be considered as second-line treatment in nonsplenectomized patients [10]. Current evidence on the use of romiplostim in adults with ITP has demonstrated rapid and sustained platelet increases while reducing the use of concomitant medications and the incidence of bleeding [9]. Currently, it is dosed weekly to maintain platelet counts >30 × 10^9^/L (in International terms >30,000/*µ*L). Usual starting dose is 1 *µ*g/kg weekly, though some centers have been able to safely start patients on 2-3 *µ*g/kg per week. Vials of romiplostim are only available in 250 *µ*g and 500 *µ*g sizes; titration by weight often involves discarding portions of these vials to meet exact dosing.

We report three cases of patients with chronic ITP who have maintained stable platelet counts >30 × 10^9^/L on biweekly dosing of romiplostim. The treatment of these three patients was started with a weekly injection, and the dose was escalated until a titrated dose was achieved that maintained platelet count >30 × 10^9^/L. Patients were then switched to a biweekly schedule and were given a rescue dose (and steroids/IVIg) if platelet counts fell to below < 30 × 10^9^/L. The characteristics and outcomes of these patients are presented.

## 2. Methods

### 2.1. Study Design

This was a retrospective case series analysis of three patients with chronic ITP who were seen at the Ottawa Hospital (Ontario, Canada). These patients were subsequently followed in a community hematology clinic. Data were collected from electronic medical records for patients with chronic ITP, with treatment refractory disease and receiving romiplostim as a part of their therapy. Demographic and disease characteristics, including time of ITP diagnosis, previous ITP treatments, and concomitant ITP treatments, were recorded. This study was approved by the OHSN-REB (Ottawa Health Science Network Research Ethics Board), and written consent was provided by patients.

### 2.2. Definitions

Guidelines established by the American Society of Hematology (ASH) define a clinical response as a sustained platelet count ≥30 × 10^9^/L and a complete response with a platelet count ≥100 × 10^9^/L [1]. Further, it defines refractory ITP as severe ITP that persists after splenectomy or patients who respond temporarily to corticosteroid therapy or IVIg [1].

### 2.3. Case Presentations

We describe three cases of patients who were able to safely maintain their platelet counts on biweekly romiplostim (Table 1).

#### 2.3.1. Case 1

**Figure 1:**
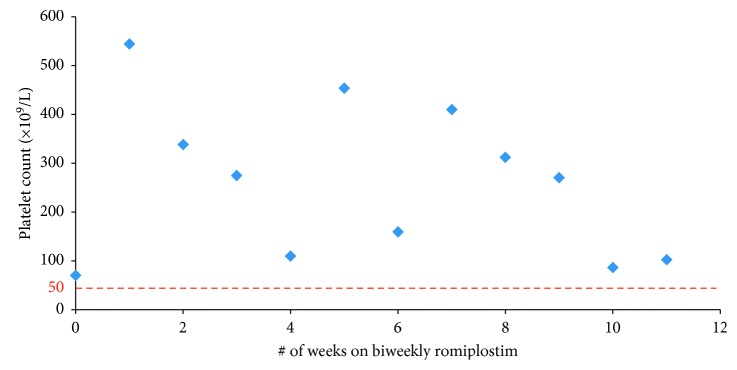
Case 1: biweekly romiplostim.

A 62-year-old female diagnosed with ITP after presenting with persistent epistaxis, thrombocytopenia, and wet purpura at age 51; she was known to have a prior history of warm autoimmune hemolytic anemia (although this was stable). She was considered to have Evan's syndrome after her ITP diagnosis. Her other comorbidities included diabetes mellitus (type II) and developmental delay. Over the next 3 years, she had frequent relapses of her ITP requiring hospitalization for epistaxis. She underwent splenectomy within the first 3 months of her ITP diagnosis, and eventually received eight courses of rituximab, multiple courses of IVIg and prednisone, and finally was started on romiplostim three years after her splenectomy. She maintained a stable platelet count on romiplostim 500 *µ*g weekly for 53 weeks. Due to platelet counts remaining in the 200–600 × 10^9^/L range, she was switched to biweekly dosing of romiplostim 250 *µ*g and was able to maintain stable platelet counts for 11 consecutive weeks (Figure 1). She experienced a mild respiratory infection after the 11^th^ week mark which caused her platelet counts to fall. She received dexamethasone and IVIg as a rescue medication and eventually modified her romiplostim dosing schedule to alternate week dosing of romiplostim 250 *µ*g and 500 *µ*g. While on biweekly romiplostim, she experienced no bleeding complications. However, given her cognitive issues, she felt weekly dosing a preferable option. Presently, her platelets remain in the 200 to 300 × 10^9^/L range while on weekly doses of romiplostim, (presently at 230 *µ*g a week).

#### 2.3.2. Case 2

**Figure 2:**
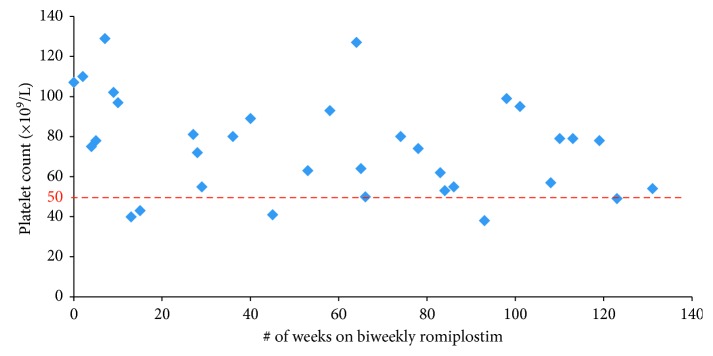
Case 2: biweekly romiplostim.

A 65-year-old female was diagnosed with chronic severe thrombocytopenia at the age of 59. She had a number of comorbidities including diabetes mellitus (type II, poorly tolerant of steroids), chronic iron deficiency, obesity, and nonalcoholic steatohepatitis. She was initially put on intermittent IVIg therapy, with platelet levels increasing from 20–30 × 10^9^/L to over 200 × 10^9^/L. The patient was not a candidate for splenectomy. The patient was started on romiplostim therapy at an initial dose of 100 *µ*g weekly and was able to maintain stable platelet counts for 38 weeks. Due to cost and convenience, a trial of biweekly dosing of romiplostim was initiated. The patient was able to maintain stable platelet counts for 131 consecutive weeks; however, due to a lapse in private medication coverage, the patient discontinued romiplostim altogether (Figure 2). Six weeks after her last romiplostim dose, she was given 4 doses of rituximab to maintain her platelet counts >30 × 10^9^/L. Currently, she is on no treatment for ITP and is in partial remission, maintaining platelet counts in the range of 37–69 × 10^9^/L. While on biweekly romiplostim, she experienced no bleeding complications.

#### 2.3.3. Case 3

**Figure 3:**
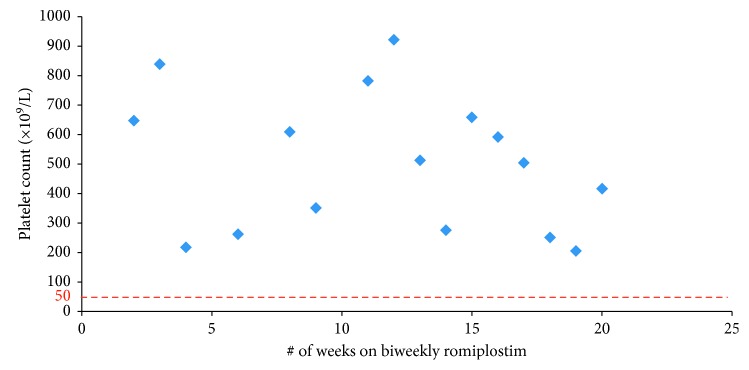
Case 3: biweekly romiplostim.

**Figure 4:**
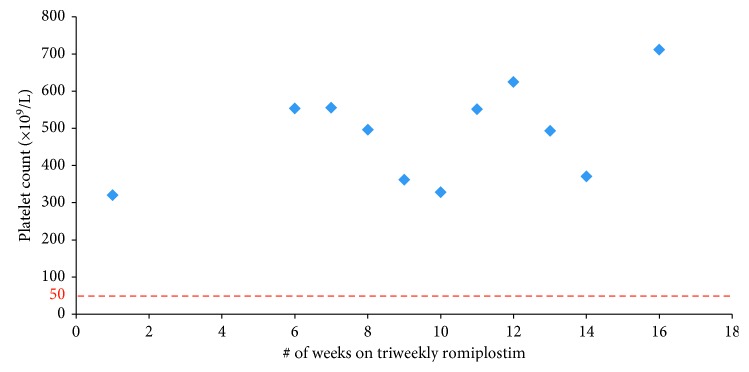
Case 3: triweekly romiplostim.

A 52-year-old female was diagnosed with chronic refractory ITP at the age of 46. She had a number of comorbidities including osteoporosis and type I diabetes mellitus. She was initially able to maintain a stable platelet count on prednisone 50–70 mg therapy for 2 years; however, due to her diabetes, she was weaned off prednisone. She underwent a splenectomy three years after her initial presentation; however, her platelet count remained under 10 × 10^9^/L one-week postprocedure. She received multiple doses of IVIg and low dose prednisone to maintain her platelet count above 30 × 10^9^/L. Romiplostim was initiated 13 months postsplenectomy. She was started on weekly romiplostim 75 *µ*g therapy. She maintained stable platelet counts on weekly romiplostim dosing for 94 weeks before being switched to biweekly romiplostim 75 *µ*g therapy and was able to maintain stable platelet counts for 20 weeks (Figure 3). She had extremely high platelet counts on biweekly romiplostim (400–700 × 10^9^/L range) allowing a trial of triweekly romiplostim dosing to be introduced. On q3weekly dosing, she was still able to maintain high platelet counts for 12 weeks (Figure 4), and thus, romiplostim therapy was discontinued altogether while monitoring the patient closely. The patient maintained a durable remission three years after her last dose of romiplostim. Like the other two patients, she experienced no bleeding complications while on biweekly dosing of the drug.

## 3. Discussion

In this paper, we report three cases of adult patients with chronic ITP who were administered biweekly dosing of romiplostim after achieving stable platelet counts (often with platelet counts well above 400 × 10^9^/L). Romiplostim, a TPO-receptor agonist, is a highly effective treatment for patients with chronic ITP following splenectomy or in patients who are not candidates for splenectomy and have failed one other form of treatment. Dosing is typically weekly via subcutaneous injection. Withholding the drug for a week is recommended if a patient's platelet count is above 400 × 10^9^/L [7]. In some instances, withholding weekly dosing of the medication (despite platelet counts over 400 × 10^9^/L) can cause a paradoxical worsening of thrombocytopenia [7]. There is a paucity of the literature regarding the clinical use of biweekly dosing of romiplostim. However, a recent study by Park et al. did address this issue in patients with acute ITP [11]. While platelet counts remained labile in patients on biweekly dosing, no bleeding complications occurred in patients on this dosing schedule.

In this case series, we retrospectively evaluated the efficacy and safety of biweekly romiplostim therapy in three patients with chronic ITP, who had been previously stable on weekly therapy. Despite the differential characteristics of the described patients (Table 1), platelet responses were rapidly observed after romiplostim therapy (1-2 weeks) in all cases. All 3 patients had maintained stable platelet counts on weekly romiplostim for at least 38 weeks prior to being switched to the biweekly dosing schedule. All three patients maintained a platelet count >30 × 10^9^/L while on biweekly romiplostim therapy (Table 2). Park et al. previously reported that a biweekly romiplostim schedule was not effective in producing a stable platelet response in patients with chronic ITP [11]. However, the three patient cases presented in this paper suggest otherwise. Furthermore, Park et al. reported that a rapid drop in platelet count was observed in all cases shortly after switching to a biweekly dosing schedule [11]. However, in the three case studies being reported, no such rapid drop in platelet count was observed. This may be due to a longer duration of stability on weekly dosing (9 months versus 3 months in the Park study) before transitioning to biweekly dosing.

In another study by Sekeres et al., patients with myelodysplastic syndrome were administered either weekly or biweekly romiplostim [12]. Durable responses were attained in patients administered weekly or biweekly romiplostim, and no differences in adverse outcomes were noted between the two groups. This study also noted that thrombocytopenic patients do not need to achieve normal platelet levels to derive clinical and quality-of-life benefit from this therapeutic intervention [12]. The goal was to achieve platelet levels that obviated the need for transfusions and lowered the risks of spontaneous or traumatic bleeding [12].

Romiplostim is administered by subcutaneous injections in a dose based on a patient's body weight and platelet count. Romiplostim, however, is only available in two doses in prefilled vials, 250 *µ*g and 500 *µ*g, costing approximately $882.50 and $1765 Cdn, respectively [13]. The dose is potentially administered indefinitely for patients with chronic ITP. At the present time, in the Canadian health care system, the yearly medication costs between $45,890 Cdn (for the 250 *µ*g weekly dose) and $91,780 Cdn (500 *µ*g weekly dose). However, certain patients, such as those presented in this case series report, can maintain stable platelet counts while on a biweekly dosing of romiplostim. On a biweekly dosing schedule, patients are able to save between $22,945 and $45,890 Cdn annually.

Interestingly, one patient (Case 3) achieved clinical remission after discontinuation of romiplostim. Several other studies have demonstrated sustained remission and a positive safety profile after discontinuing romiplostim, mainly in chronic relapsing or refractory ITP patients [14–17]. The mechanisms and factors of remission in patients with ITP remain unknown, although some studies suggest a restoration of immune tolerance and a decrease of inflammatory state after continuous treatment with TPO-receptor agonists through the stimulation of regulatory B and T lymphocytes [18, 19]. We conclude that if a patient does appear to be entering a remission from ITP while on romiplostim, there may be a role for attempting biweekly dosing as part of a potential tapering strategy.

The half-life of romiplostim is estimated to be from 1 to 34 days, suggesting that lengthening the interval of romiplostim administration to more than a week may be possible in some patients, and should cautiously be evaluated on a case-by-case basis [8, 11, 20, 21]. Early pharmacokinetic mathematical modeling studies further lend support to this possibility; weekly romiplostim dosing has a more predictable platelet count profile, but biweekly dosing can still lead to acceptable platelet counts, above the minimum requirements of 30 × 10^9^/L [21]. The maintenance of stable platelet counts after initiating biweekly dosing of romiplostim suggests different physiological responses to romiplostim in ITP patients and highlights the importance of a close follow-up in these patients. Based on our real-life experience, a transition to biweekly romiplostim dosing may be attempted in those who have achieved clinical stability on a weekly dosing regimen for at least six months and have demonstrated platelet counts typically of 400 × 10^9^/L or greater while on weekly dosing. This is especially helpful for patients with financial challenges. Finally, the alternate week dosing may be attempted in those that may be entering a remission from their ITP with eventual goal to stop all therapies.

## 4. Conclusion

In conclusion, lengthening the dosing frequency of romiplostim to biweekly may have a role to select patients with chronic ITP. Our data suggest that a trial of biweekly dosing may be implemented in patients who have been stable on weekly dosing for at least six months and may be entering clinical remission. All three patients reported in this case series had no adverse bleeding symptoms while on biweekly romiplostim dosing; one patient eventually experienced a sustained remission from her ITP. Although romiplostim is traditionally given on a weekly schedule, based on the present findings, it might be valuable to evaluate the efficacy of biweekly dosing on a case-by-case basis. In clinical practice, required doses and intervals are expected to differ between patients and the minimal effective dose to maintain adequate platelet levels should be determined individually. Further studies are warranted to determine which patients can safely be transitioned to biweekly dosing; this will ultimately reduce the cost of the medication and increase convenience to patients.

## Figures and Tables

**Table 1:** Baseline demographic and clinical characteristics.

Characteristic	Case #1	Case #2	Case #3
Age of ITP diagnosis	57	63	46
Gender	Female	Female	Female
Weight	60 kg	120 kg	80 kg
ITP etiology	Primary	Primary	Primary
Number of prior ITP therapies	4	2	2
Prior therapies for ITP			
** **Prednisone (maintenance)	10 mg	No	No
** **IVIg (# of doses)	16 doses	4 doses	21 doses
** **Rituximab	8 doses	4 doses	No
** **Splenectomy	Yes	No	Yes
** **Vincristine	Yes	No	No
** **Eltrombopag	Yes	No	No
** **Dexamethasone	Yes	No	No

**Table 2:** Detailed summary of romiplostim dosing schedule.

Parameter	Case #1	Case #2	Case #3
Romiplostim started (postdiagnosis)	3 years (2015)	2 years	3 years
Trial 1			
** **Dose	500 *µ*g	100 *µ*g	75 *µ*g
** **Frequency	Weekly	Weekly	Weekly
** **Duration	53 weeks	38 weeks	94 weeks
** **Platelet range (× 10^9^/L)	31–722	35–221	42–595
Trial 2			
** **Dose	250 *µ*g	250 *µ*g	75 *µ*g
** **Frequency	Biweekly	Biweekly	Biweekly
** **Duration	11 weeks	131 weeks	20 weeks
** **Platelet range (× 10^9^/L)	86–454	40–129	217–922
Trial 3			
** **Dose	500 *µ*g	—	75 *µ*g
** **Frequency	Weekly	—	Triweekly
** **Duration	42 weeks	—	12 weeks
** **Platelet range (× 10^9^/L)	31–1277	—	321–625
Current ITP status	Stable^*∗*^	Partial remission	Complete remission

^*∗*^On weekly dosing of romiplostim 230 *µ*g.
